# Evaluation of hepcidin-25/erythroferrone ratio as a potential biomarker for iron utility and erythropoiesis responsiveness to erythropoiesis-stimulating therapy in comparison to immature erythrocyte/reticulocyte parameters in hemodialysis patients

**DOI:** 10.1016/j.htct.2024.04.125

**Published:** 2024-08-21

**Authors:** Salwa Bakr, Karem Mohamed Salem, Ahmed Mohammed Rashed, Mohamed E A Tantawy, Asmaa Younis Elsary, Hanan Abdelmoneam Shamardl, Eman Mahmoud Ezzat

**Affiliations:** a Clinical Pathology/ Hematology, Faculty of Medicine, Fayoum University, Fayoum, Egypt; b Internal Medicine, Faculty of Medicine, Fayoum University, Fayoum, Egypt; c Faculty of Medicine, Menoufia University, Egypt; d Public Health and Community Medicine, Faculty of Medicine, Fayoum University, Fayoum, Egypt; e Medical Pharmacology, Faculty of Medicine, Fayoum University, Fayoum, Egypt

**Keywords:** Anemia, ESRD, Hepcidin, Erythroferrone, Reticulocyte hemoglobin content, Erythropoiesis-stimulating agent

## Abstract

**Background:**

Anemia-associated chronic kidney disease increases in more advanced stages with a subsequent acceleration in renal impairment progressing to end-stage renal disease. Although hepcidin and erythroferrone have been described as novel biomarkers of iron metabolism, there is still an area of ambiguity regarding iron utility in anemia-associated end-stage renal disease.

**Objectives:**

This study aims to determine the correlations between erythropoietin, erythroferrone, and hepcidin-25 in hemodialysis, and to evaluate the clinical utility of the hepcidin-25/erythroferrone ratio as a biomarker of erythropoiesis-stimulating agent effectiveness compared to reticulocyte maturation parameters.

**Methods:**

Serum erythropoietin, erythroferrone, and hepcidin-25 levels in 35 dialysis-dependent patients on a maintenance dose of a short-acting erythropoiesis-stimulating agent were consequently assessed on Days 0, 5, and 7. The erythropoiesis activity was monitored by measuring the increment in reticulocyte maturation parameters.

**Results:**

Though the effectiveness of erythropoiesis in these patients was not associated with the hepcidin-25/erythroferrone ratio, it was lower among those with effective erythropoiesis than those with ineffective erythropoiesis. The effective group showed a statistically significant increase in reticulocyte maturation parameters compared to the ineffective group.

**Conclusions:**

The findings show the pathogenesis of iron homeostasis in hemodialysis, the validity of hepcidin-25/erythroferrone ratio as a biomarker of erythropoiesis-stimulating agent effectiveness, and the advantageous monitoring of reticulocyte maturation measures to improve management of anemia-associated chronic kidney disease.

## Introduction

Anemia-associated chronic kidney disease (CKD) is a disease that increases in more advanced stages with a subsequent acceleration in renal impairment progressing to end-stage renal disease (ESRD). It is caused by multifactorial pathogenesis of relative erythropoietin (EPO) deficiency, systemic inflammation, hepcidin-impaired clearance rate, iron restriction, nutritional deficiencies (vitamin B12 and folic acid), shorter erythrocyte lifespan, potential chronic blood loss during dialysis, and repeated phlebotomies.^1^ In CKD, anemia contributes to increases in the morbidity potential and mortality rate with significant clinical and economic burdens on healthcare systems with worse quality of life, renal function, cardiovascular performance, cognitive function, and immune function, as well as altered hemostasis.^2^

According to the World Health Organization, erythropoiesis-stimulating agent (ESA) dose requirements vary between individuals and are hard to be predicted as they are related to associated comorbidities and the intensity of inflammation. Therefore, adjustment of the ESA dose is subject to the initial hemoglobin level, the targeted level, and the observed increase. Additionally, it has been suggested that starting ESA therapy should be at a hemoglobin level between 9 and 10 g/dL, aiming to raise the level slowly at a rate of <1–2 g/dL per month. Moreover, it has been recommended that the level of hemoglobin should not exceed 11.5 g/dL during ESA therapy to avoid the risk of fatal stroke and vascular thrombosis.^3^

Erythropoiesis and iron homeostasis are reciprocally regulated and linked at multiple levels with the EPO-erythroferrone (ERFE)-hepcidin (HEP-25) axis playing a major role in iron metabolism.^4^ During erythropoiesis, in response to anemia mediated by hypoxia-inducible factor-2α or exogenous EPO administration, enhancement of ERFE synthesis, which is encoded by the FAM132B gene in erythroblasts, inhibits HEP-25 production, and thus promotes iron acquisition, storage, and utilization.^4^^,^^5^ In general, HEP-25 production by hepatocytes increases in response to inflammation and iron overload, and consequently diminishes with hypoxia and iron deficiency.^1^ In hemodialysis patients, HEP-25 levels increase significantly (even up to nine-fold levels) as a consequence of several factors including impaired renal clearance, the paradoxical administration of IV iron therapy and the intensification of subclinical inflammatory-related immune activation that might contribute to EPO resistance despite iron availability.^1^^,^^6^

Classical biomarker monitoring of hemoglobin response during ESA therapy is not very sensitive and is very much delayed, often by at least four weeks.^7^^,^^8^ Several studies have reported that the reticulocyte-hemoglobin content (RET-He) is a useful measure to evaluate functional iron deficiency long before apparent distinguishable changes in absolute increments in the circulating reticulocyte (RET) count, red cell indices and hemoglobin concentration.^9^ Moreover, the immature RET fraction (IRF) is a useful parameter to predict accelerated bone marrow recovery at an early stage.^10^

Although the HEP-25 and ERFE serum levels have been described as novel biomarkers of iron metabolism,^11^ there is still an area of uncertainty regarding their clinical utility as diagnostic tools in ESRD.^1^^,^^4^ Because the mechanisms that explain inter-individual variation in the improvement of anemia-associated CKD have still not been clarified,^11^ the present research aimed to determine correlations between circulating EPO, ERFE, and EP-25 serum levels and the effectiveness of erythropoiesis in ESRD. Additionally, it aimed to evaluate the clinical utility of the HEP-25/ERFE ratio as a biomarker of ESA effectiveness in erythropoiesis in comparison to immature erythrocyte/RET parameters (including RET-He and IRF) in hemodialysis patients after starting anemia treatment. Understanding the mechanisms that underlie the dysregulation of iron balance in chronic kidney impairment is a good indicator for developing new diagnostic tools and therapeutic strategies to improve the management of anemia-associated CKD.^4^^,^^6^

## Material and methods

### Study design

This prospective cohort study enrolled 35 over 18-year-old hemodialysis patients as per the ethical standards of the Declaration of Helsinki and after receiving the approval of the local ethics committee of the institution. This study was carried out in the dialysis unit of Fayoum University Hospital and in two non-profit dialysis units located in two different Egyptian governorates.

### Patient population

#### Inclusion and exclusion criteria

This prospective cohort study included patients with ESRD on an adequate dialysis rate using EPO at a maintenance dose for at least the previous three months.

Patients with infections, nutritional anemia (iron, folate, or B12), or chronic inflammatory diseases, such as autoimmune disease, severe lung or liver diseases, malignancies, and those who received anti-inflammatory or immunosuppressive drugs, ingested iron medications or received adjuvant therapy within the previous month were all excluded from the study.

### Patient classification

Detailed history taking, clinical examination, and laboratory investigation (including high-sensitivity C-reactive protein, complete blood counts (CBC) with RET parameters, iron profile, creatinine level, dialysis vintage, parathyroid hormone (PTH), serum phosphorus, serum calcium, random serum blood sugar, liver enzyme (ALT), and serum level of vitamin D) were assessed for all participants. Moreover, the EPO resistance index (ERI) was estimated (mean ESA per IU/kg/Hb over a three-month period).^12^

Additionally, serial quantitative assessments of circulating EPO, ERFE, and HEP-25 serum levels were made on Days 0, 5, and 7 after injecting a short-acting ESA. Marrow erythropoiesis activity of each patient was evaluated by comparing erythrocyte and RET values (particularly the IRF and RET-He levels) before and after the injection of ESA on Days 0 and 7. Patients were allocated to effective or ineffective erythropoiesis groups based on the increase in the RET-He value after ESA injections. Accelerated, but ineffective, erythropoiesis was noted among patients who had an increase in IRF without the obvious increment in the RET-He levels after the injection of ESA.

#### Blood sampling and laboratory testing

Peripheral blood samples collected from each study patient (4 mL) were equally divided into two tubes - one containing ethylenediaminetetraacetic acid (EDTA) – after receiving written informed consent from the patient.

### Estimation of marrow erythropoiesis activity

Blood samples in EDTA were analyzed within two hours of collection using the Sysmex CX 1000 blood analyzer with Cellpack-DFL™ diluent and Fluorocell™ RET dye reagents in the RET channel (Sysmex Corporation, Kobe, Japan) to assess the CBC with RET parameters. These parameters included: the absolute RET value (RET#), RET percentage (RET%), RET-He, IRF, and percentages of high fluorescent RET (HFR%), middle fluorescent RET (MFR%), and low fluorescent RET (LFR%).

### Quantitative detection of serum erythropoietin, hepcidin-25, and erythroferrone

Serum samples were left to stand at room temperature until the separation of the serum by centrifugation at 4 °C at a speed of 3000 rev/min. The separated sera were stored at −20 °C until analyses. It was then thawed for quantitative testing of EPO, ERFE, and HEP-25 using an enzyme-linked immunosorbent assay (ELISA) sandwich kit from the Bioassay Technology Laboratory (BT Lab, Shanghai, China) as per the manufacturer's instructions.

### Statistical analysis

Data analysis was performed using the statistical package of social science (SPSS) software version 22 in Windows 7 (SPSS Inc., Chicago, IL, USA). The analysis included a simple descriptive analysis in the form of numbers and percentages of qualitative data with arithmetic means as central tendency measures and standard deviations as a measure of the dispersion of quantitative parametric data. The Mann-Whitney test was used to compare two independent groups. The Wilcoxon test was used to compare two groups of dependent data. The Chi-square test was used to compare two or more groups of qualitative data, whereas the Pearson bivariate correlation was used to test associations between variables. P-values <0.05 were considered statistically significant.

## Results

The present study enrolled 35 hemodialysis patients with a mean age of 55.5 ± 12.2 years and a mean body mass index (BMI) of 28.4 ± 3.2 kg/m^2^. The mean dialysis vintage was 58.6 ± 46.5 min. Almost half (51.4 %) of the study group were males. The majority of the study population were married (77.2 %) and non-smokers (82.9 %). The most common cause of CKD was hypertension (45.7 %). The study results show that after adequate dialysis and ESA (epoetin alpha) injections (4000–12,000 IU) at a weekly injection interval, the outcomes of erythropoiesis were effective in 54.3 % patients, ineffective in 34.3 % and accelerated but ineffective in 11.4 % of the cases (Table 1).

**Table 1:** Description of demographic and clinical characteristics of the patients.

**Variable**		**Number** (*n* = 35) Mean ± SD	

Age (years)		55.5 ± 12.2	
BMI (kg/m^2^)		28.4 ± 3.2	
Dialysis vintage (m)		58.6 ± 46.5	
Duration of DM (years)		17.6 ± 9.2	
Duration of HTN (years)		12.7 ± 8.7	


**Blood pressure** (mmHg)			
Pre-dialysis	Systolic	123.7 ± 18.5	
	Diastolic	77.3 ± 12.2	
Post-dialysis	Systolic	122.6 ± 20	
	Diastolic	76.2 ± 11.7	

		n	%

**Gender**			
Male		18	51.4
Female		17	48.6
**Smoker**			
No		29	82.9
Yes		6	17.1
**Co-morbidities**			
Hypertension		27	77.2
Diabetes mellitus		12	34.3
Cardiac disease		9	25.7
Respiratory disease		3	8.6
Rheumatologic disorders		1	2.9
Hepatic disease		0	0
Malignancy		0	0
**Original kidney disease**			
HTN		16	45.7
Polycystic kidney, stones		5	14.3
DM & HTN		4	11.4
DM		3	8.6
Unknown		3	8.6
Stones & HTN & DM		2	5.7
Stones		1	2.9
Congenital abnormality		1	2.9
**Erythropoiesis Outcome**			
Ineffective		12	34.3
Effective		19	54.3
Accelerated but ineffective		4	11.4

BMI: body mass index: DM: diabetes mellitus; HTN: hypertension.

There were no statistically significant differences (p-value >0.05) between patients with effective and ineffective outcomes in respect to basic characteristics (co-morbidities, antihypertensive medications, and the ERI - Table 2), and for routine lab results. Although there was no significant difference (p-value = 0.6) in the HEP-25/ERFE ratio related to outcomes of erythropoiesis, the ratio was lower among patients with effective rather than those with ineffective erythropoiesis (Table 3).

**Table 2:** Comparison between erythropoiesis outcome and clinical characteristics of patients.

**Variable**	**Ineffective** (*n* = 16)		**Effective** **(*n*=19)**		**p-value**
	**Mean**	**SD**	**Mean**	**SD**	
**Age** (years)	54.9	10.9	56.2	13.4	0.7
**BMI** (kg/m^2^)	28.3	3.1	28.6	3.4	0.8
	**Median**	**Range**	**Median**	**Range**	
**Dialysis vintage** (m)	48	24–120	36	7–120	0.9
**Duration of DM** (years)	18	9–32	19	2–25	0.8
**Duration of HTN** (years)	15	10–30	12.5	3–15	0.8
ERI*	8.4	3.7–14.1	7.7	4.2–12.8	0.8
	**n**	**%**	**No.**	**%**	
**Gender**					
Female	7	43.8 %	10	52.6 %	0.7
Male	9	56.3 %	9	47.4 %	
**Smoking**					
No	13	81.3 %	16	84.2 %	0.9
Yes	3	18.8 %	3	15.8 %	
**ESA dose (IU/week)**⁎⁎					
4000	6	37.4 %	7	36.8 %	0.5
8000	9	56.3 %	12	63.2 %	
12,000	1	6.3 %	0	0 %	
**Co-morbidities**					
Hypertension	13	81.3 %	14	73.7 %	0.7
Diabetes mellitus	5	31.3 %	7	36.8 %	0.9
Cardiac disease	4	25 %	5	26.3 %	0.9
Respiratory disease	0	0 %	3	15.8 %	0.2
Rheumatologic disorders	1	6.3 %	0	0 %	0.5
Hepatic diseases0	—	—	—	—	—
Malignancy	—	—	—	—	—
**Antihypertensive medication**					
No	5	33.3 %	7	36.8 %	0.9
ACEI/ARBs	3	20 %	4	21.1 %	
Non-ACEI/ARBs	7	46.7 %	8	42.2%	
**Vitamin D intake**					
No	13	81.3 %	13	72.2 %	0.7
Yes	3	18.7 %	5	27.8 %	
**Other medications**					
No	10	62.5 %	15	78.9 %	0.4
Yes	6	37.5 %	4	21.1 %	

DM: diabetes mellitus; HTN: hypertension; ERI: erythropoietin resistance index; ACEI: angiotensin-converting enzyme inhibitors; ARBs: angiotensin II receptor blockers.

⁎ Erythropoietin Resistance Index (ERI) = ESA per kg/Hb level.

⁎⁎ Epoetin alpha prescribed at a weekly injection interval.

**Table 3:** Comparisons of laboratory results of the different study groups.

**Variable**	**Ineffective** (*n* = 16)		**Effective** **(*n*=19)**		**p-value**
	**Mean**	**SD**	**Mean**	**SD**	
Hemoglobin in last 3 months (g/dL)	11.7	1.5	11.6	1.3	0.9
Hemoglobin in last 2 months (g/dL)	10.9	0.76	10.9	1.01	0.9
Hemoglobin in last months (g/dL)	10.5	0.77	10.9	1.2	0.5
Serum Creatinine (mg/dL)	8.9	1.7	9.4	2.3	0.4
Urea Reduction Ratio (URR) (%)	70.4	3.3	70.9	4.7	0.7
PTH (pg/mL)	135.4	107.8	144.4	163.1	0.8
Total Calcium (mg/dL)	8.6	0.81	9	1.1	0.3
Phosphorous (mg/dL)	5.2	1.7	4.5	1.3	0.2
Serum Iron (µg/dL)	80.3	21.9	98.02	39.7	0.1
TIBC (U/dL)	256.9	52.9	234.1	82.8	0.3
Serum Ferritin (µg/L)	542.3	437.7	703.8	461.8	0.3
CRP (mg/dL)	9.7	8.1	7.1	5.2	0.2
ALT (IU/L)	14.5	2.8	13.4	2.4	0.2
Serum HEP-25/ERFE ratio	151.7	58.1	141.8	38.8	0.6


**Virology**	**No**	**%**	**No**	**%**	
HBVs-Ag (+ve)	0	0 %	0	0 %	—
HCV-Ab (+ve)	1	6.3 %	3	15.8 %	0.6
HIV-Ab (+ve)	0	0 %	0	0 %	—

PTH: parathyroid hormone; TIBC: total iron binding capacity; CRP: C-reactive protein; ALT: alanine transaminase; HEP-25: hepcidin; ERFE: erythroferrone; HBVs-Ag: hepatitis B virus surface antigen; HCV-Ab: hepatitis C virus antibodies; HIV-Ab: Human immune deficiency virus antibodies.

In this study, there was a statistically significant difference (p-value = 0.04) between the ineffective and effective groups on Day 0 as regards RET-He, with a lower median for the effective group (26.7 versus 30.9). There was also a statistically significant difference (p-value = 0.04) between ineffective and effective groups on Day 7 as regards RET% and RET#, with higher medians for the effective group (Table 4). The ineffective group showed a statistically significant five-fold increase in serum EPO (p-value = 0.02) after seven days of ESA injections with a statistically significant decrease in RET% (p-value = 0.002). Regarding the effective group, there were significant increases in RET%, RET#, RET-He, IRF, LFR, MFR, and HFR levels after seven days of epoetin alpha injections (p-values = 0.004 and 0.001 - Table 4).

**Table 4:** Comparisons of sequential serum levels of EPO, ERFE and HEP-25 and erythrocyte and reticulocyte parameters at different timepoints.

Variable	Day 0	Day 5	Day 7	P-value⁎⁎
	Median (range)	Median (range)	Median (range)	
**Serum EPO**				
Ineffective	84.7 (60.8–617.2)	443.9 (101.2–608.3)	452.8 (77.8–589.7)	**0.02**⁎⁎
Effective	84.1 (63.6–614.3)	86.9 (68.5–598.4)	89.2 (64.6–594.7)	0.95
p-value	0.781	0.57	0.46	
**Serum ERFE**				
Ineffective	3.5 (1.5–25.9)	16.2 (3.5–26.2)	17.3 (2.6–25.4)	0.17
Effective	2.7 (2–26.7)	2.9 (1.8–25.8)	2.9 (1.6–25.2)	0.96
p-value #	0.75	0.44	0.68	
**Serum HEP-25**				
Ineffective	440.8 (133.7–3657.9)	2197.4 (307.1–3508.2)	2746.4 (189.1–3779.7)	0.35
Effective	405.2 (184.2–3580.1)	423.7 (305.7–3953.4)	500 (267.4–3667.9)	0.75
**P-value** #	0.71	0.64	0.83	
**RBCs**				
Ineffective	3.7 (2.9–4.9)	——	3.88 (3.2–5.1)	0.19
Effective	3.8 (3.2–5.8)	——	4.1(3.4–5.4)	0.37
p-value #	0.85	——	0.48	
**HB** (g/dl)				
Ineffective	10.8 (8.3–12.9)	——	10.2 (9–12.8)	0.09
Effective	10.5 (9.6–12.3)	——	11.2 (10.3–12.3)	0.14
p-value #	0.88	——	0.23	
**RET%**				
Ineffective	1.7 (1.2–3.1)	——	1.3 (1.1–1.6)	**0.002**⁎⁎
Effective	1.8 (0.75–2.84)	——	2.3 (1.5–3.03)	**0.004**⁎⁎
p-value #	0.83	——	0.003*	
**RET#**				
Ineffective	0.08 (0.04–0.21)	——	0.05 (0.03–0.91)	0.05
Effective	0.07 (0.03–0.11)	——	0.10 (0.07–0.20)	**0.001**⁎⁎
p-value #	0.22	——	0.004*	
**RET-He**				
Ineffective	30.9 (27.9–36.5)	——	31(28.3–33.5)	0.06
Effective	26.7 (20–34.7)	——	31.9 (26.4–36.9)	**0.001**⁎⁎
p-value #	0.04*	——	0.44	
**IRF**				
Ineffective	13.9 (9.1–29.9)	——	11.9 (6.1–16.4)	0.13
Effective	10.8 (1.1–29.1)	——	16 (7.4–25.1)	**0.001**⁎⁎
p-value #	0.07	——	0.15	
**LFR**				
Ineffective	86.2 (29.8–90.9)	——	88.1(83.6–93.9)	0.16
Effective	89.2 (70.9–98.9)	——	84 (75–92.6)	**0.001**⁎⁎
p-value #	0.07	——	0.17	
**MFR**				
Ineffective	10.9 (7.3–15.2)	——	10 (5.2–13.5)	0.51
Effective	9 (1–16)	——	12 (7.2–15.2)	0.004**
p-value #	0.19	——	0.26	
**HFR%**				
Ineffective	3.1(1–15.6)	——	1.9 (0.9–3)	0.21
Effective	1.4 (0.1–13.1)	——	2.8 (0.2–9.8)	**0.001**⁎⁎
p-value #	0.07	——	0.32	

# significance between ineffective and effective groups.

⁎⁎ significance between Day 0 and Day 7 in each group. EPO: erythropoietin; ERFE: erythroferrone; HEP-25: hepcidin; RBCs: red blood cells; Hb: hemoglobin; RET%: reticulocyte percentage; RET#: absolute value of reticulocytes: RET-He: reticulocyte hemoglobin content; IRF: immature reticulocyte fraction; LFR: low fluorescent reticulocyte; MFR: middle fluorescent reticulocyte; HFR: high fluorescent reticulocyte.

An analysis of the correlation of HEP-25 serum levels with EPO and ERFE (Day 7) showed there were statistically positive correlations (*r* = 0.96 and 0.97, respectively; p-values <0.001) both for the effective group (*r* = 0.97 and 0.97, respectively; p-values <0.001) and for the ineffective group (*r* = 0.97 and 0.98, respectively; p-value <0.001. In addition, there was a positive significant correlation between circulating ERFE and EPO serum levels on Day 7 (*r* = 0.99; p-value <0.001) in both effective and ineffective groups with *r* = 0.98 and *r* = 0.99, respectively (p-value <0.001). However, intra-individual serum levels of both EPO and HEP-25 on Days 5 and 7 were much higher than on Day 0 among those individuals in the ineffective group unlike those in the effective group (Table 4).

Using multiple linear regressions, the effectiveness of erythropoiesis (increment in RET-He level on Day 7) was associated with the red cell count and hemoglobin level (*r* = 0.78; p-value 0.001 and *r* = 0.61; p-value 0.02, respectively), but was not associated with the HEP-25/ERFE ratio.

On studying the EPO resistance index (ERI) using RET-He (ESA UI/kg/RET-He) instead of hemoglobin level (ESA UI/kg/Hb), the study showed a statistically significant positive correlation (*r* = 0.84; p-value = 0.001).

## Discussion

In the last two decades, understanding the cross-talk between biomarkers of iron metabolism and erythropoiesis has yielded significant advances.^6^ During erythropoiesis in healthy individuals, erythroblasts produce ERFE which regulates HEP-25 production, and thus, iron metabolism.^5^ Nevertheless, studies on ERFE in CKD patients (particularly in those under dialysis) are limited with slightly conflicting results.^6^ Studies conducted on CKD so far have revealed that serum ERFE concentration rises within 24 h for approximately seven days in response to a single dose of ESA exhibiting a circadian rhythm,^13^, ^14^, ^15^ while the correlation between serum ERFE and HEP-25 levels remains ambiguous and unclear.^1^ Furthermore, the associations between circulating ERFE in CKD patients and the biomarkers of erythropoiesis have not been evaluated.^1^^,^^5^^,^^13^ Hence, the present study enrolled 35 anemic hemodialysis patients on short-acting ESA (epoetin alpha), which was usually administrated two or three times weekly, aimed to determine correlations between circulating EPO, ERFE, and HEP-25 with erythropoiesis. In addition, it aimed to evaluate the clinical utility of the HEP-25/ERFE ratio as a marker of erythropoiesis compared to immature RET parameters (RET-He and IRF).

In 2021, the Kidney Disease Improving Global Outcomes (KDIGO) guidelines suggested updating anemia guidelines to use the RET-He biomarker in the clinical practice to assess marrow efficiency of erythropoiesis at an early stage.^16^ Furthermore, the nephrology clinical practice guidelines of the National Kidney Foundation - Kidney Disease Outcomes Quality Initiative (NKF KDOQI) reported that RET-He is a sensitive early marker for assessing the bioavailability of iron for erythropoiesis^17^ Therefore, monitoring increments of RET-He was selected in this study as a biomarker indicator of the effectiveness of erythropoiesis.

The results of the present study found that the outcome of erythropoiesis after adequate dialysis (urea reduction ratio >65 %) and administration of epoetin alpha (4000–12.000 IU weekly) at frequent injection intervals were effective in 54.3 % of cases, ineffective in 34.3 %, and accelerated but ineffective in 11.4 %. That is despite the fact that there were no significant differences between patients with effective and ineffective outcomes regarding basic characteristics, such as ESA dose, ERI, co-morbidities, antihypertensive medications and routine lab results.

On studying the correlations of the HEP-25 serum level with EPO and ERFE on Day 7, there were statistically positive differences both for the effective group (*r* = 0.97 and 0.97, respectively; p-value <0.001) and the ineffective group (*r* = 0.97 and 0.98, respectively; p-value <0.001). In addition, there was a positive significant correlation between serum EPO and ERFE levels on Day 7 in both effective and ineffective groups (*r* = 0.98 and *r* = 0.99, respectively; p-value <0.001). This is in agreement with the two published studies on ERFE in patients with CKD. The studies noted that circulating ERFE showed a dose-response relationship with the amount of ESA administered.^5^^,^^13^ However, intra-individual serum levels of both EPO and HEP-25 on Day 5 and Day 7 in this cohort were much higher than on Day 0 for individuals in the ineffective group compared to the effective group. Honda et al. described a similar result that ERFE in hemodialysis patients correlated positively with the circulating EPO level but did not correlate with HEP-25. This pathological modulation of HEP-25, which might occur due to the inflammation linked to renal disease in addition to impaired clearance, could explain the HEP-lowering effect of ERFE in CKD.^6^ Moreover, the results of this study showed that most of the patients with effective erythropoiesis (63.2 %) were on a maintenance dose of epoetin alpha (8000 IU/week administrated as two doses). Though there was no statistically significant difference between the two studied groups, a statistically positive correlation of serum ERFE levels between Day 0, Day 5, and Day 7 was observed intra-individually even among the patients with ineffective erythropoiesis. This is in agreement with the finding regarding the effectiveness of prescribing short-acting epoetin alpha with longer injection intervals of Spinowitz et al. Their study, enrolled a cohort of non-dialysis patients with anemia of CKD and used variable doses of short-acting ESA (epoetin alpha) (10.000, 20.000, and 40.000 IU) at different intervals (1, 2 and 4 weeks). The authors reported that the pharmacodynamic effect of epoetin alpha on erythropoiesis response does not appear to be allied to the drug half-life or the interval of the dose but rather to the erythrocyte lifespan. It was seen in this study that despite the short serum half-life of epoetin alpha (24–30 h), its subcutaneous administration in patients with CKD could effectively increase the level of hemoglobin concentration when injected at intervals of up to four weeks. The authors suggested that once an ESA injection is administrated, maturation of available hematopoietic precursor cells initiates then over time, they enter blood circulation with an increase in hemoglobin concentration over a period of weeks.^18^ In accordance with a study which was conducted on 300 hemodialysis patients, Hanudel et al. and Ganz et al. reported that the duration of hemodialysis sessions correlated with the ESA response where the addition of one extra hour could diminish the ESA dose by nearly 2000 IU/week.^13^^,^^14^^,^^19^

The present study clearly showed that the serum EPO levels on Days 5 and 7 were much higher with an up to a five-fold increase among individuals in the ineffective group rather than those in the effective group. This suggests the possibility of an EPO hyporesponsiveness in the ineffective group. However, there was no statistical significant difference between the ERI and effectiveness of erythropoiesis in the current cohort which might be explained by a small sample size of the study population. Resistance to ESA therapy or ESA hyporesponsiveness that occurs when CKD patients do not achieve the target hemoglobin level (even with higher-than-usual doses of an ESA agent or when increasingly higher doses are required to maintain the desired hemoglobin level)^20^ can raise the risk of negative outcomes related to increased blood viscosity, hypertension, and cardiovascular risk.^21^ Therefore, avoiding repeated escalations of ESA doses beyond double the initial weight-based dose was recommended in the KDIGO guidelines.^22^ In fact, ESA therapy results in effective erythropoiesis as a consequence of HEP-25 suppression and releasing stored iron,^5^ and the outcomes in the responsiveness of marrow erythrocyte progenitor cells in dialysis-dependent patients treated with ESAs are inversely related to the amount of circulating cytokines as well as the severity of chronic inflammatory disease.^1^ In a prospective cohort study that enrolled 12,389 patients with ESRD in 21 countries, a prompt decrease in hemoglobin levels along with the increase in ESA doses was observed among patients with an increased level of C-reactive protein parallel to a rise in the incidence of ESA therapy resistance with poor disease outcome and high mortality.^4^^,^^21^ Although the mechanism of ESA hyporesponsiveness in hemodialysis patients is not yet fully understood, the malnutrition-inflammation status could be a contributing factor.^23^^,^^24^ On probable inadequate dialysis, the HEP-inhibitory effect of ERFE might be blunted.^25^ Additionally, the classical pathogenesis of anemia in dialysis-dependent patients is usually associated with excessive PTH secretion and secondary hyperparathyroidism (HPTS) with subsequent fibrosis of bone marrow that could interfere with erythropoiesis and increase the required ESA dose.^1^ Therefore, it is essential to individualize an anemia management protocol in ESRD patients that is based on selective recognition and appropriate intervention for the potential causes of resistance in each patient before proposing an increased ESA dosage.^1^

On using multiple linear regression analysis, the effectiveness of erythropoiesis was not associated with the HEP-25/ERFE ratio in this study cohort. Although the ratio was lower among those with effective rather than ineffective erythropoiesis, there was no significant difference (p-value = 0.6). This finding is unlike that of Hara et al. who enrolled hemodialysis patients with iron deficiency after the administration of ferric citrate hydrate. The study revealed a significant inverse correlation between serum ERFE and HEP-25 levels, where the low HEP-25/ERFE ratio was only exhibited among individuals who displayed significantly increased hemoglobin levels at three months.^11^

The results of the current study, showed that there was a statistically significant difference in the increment of RET-He (p-value = 0.04) between ineffective and effective groups on Day 7 as regards RET% and RET# with a higher median in the effective group. Furthermore, there were significant increases in the RET%, RET#, RET-He, IRF, LFR, MFR, and HFR levels in the effective group on Day 7 of ESA therapy (p-values = 0.001 and 0.004). As for the ineffective group, it showed a statistically significant decrease in RET% after seven days of ESA therapy (p-value = 0.002). Furthermore, a blunted response in the level of RET-He was observed among the patients having ineffective erythropoiesis outcomes (p-value = 0.06). This indicates the potential of EPO resistance, unlike the patients with effective erythropoiesis (p-value = 0.001). Previous studies in concordance with this study revealed that RET-He could be used as an alternative parameter for diagnosing iron deficiency anemia in regular dialysis-dependent patients.^26^, ^27^, ^28^, ^29^, ^30^ when Early dose adjustment to reduce the risks associated with inappropriately high doses of EPO was calculated using RET-He (ESA UI/kg/RET-He) in the current cohort instead of the classic calculation using hemoglobin concentration (ESA UI/kg/Hb), there was a statistically significant positive correlation with the ERI (*r* = 0.84; p-value = 0.001). This is in response to the recommendation of Xu et al. for the urgency to find a sensitive early biomarker for early identification of ESA-resistance in patients instead of waiting to calculate the ERI using the increase in hemoglobin concentration. This is because calculations using the ERI method are highly delayed; they take at least four weeks with potential harm to patients with inappropriate dose escalation.^15^

Findings in this study add to the understanding of the pathogenesis of iron homeostasis in ESRD and elucidate the validity of the HEP-25/ERFE ratio as a biomarker of ESA effectiveness.

The limitation of this study is that, despite its multicenter nature, the cohort involved Egyptian patients only. Thus, it is uncertain whether the results can be generalized to other ethnic groups with ESRD.

## Conclusion

The current study sheds new light on the validity of the HEP-25/ERFE ratio as a biomarker for iron utility and erythropoiesis responsiveness to ESA in CKD patients on hemodialysis. Additionally, it is a good indicator in the monitoring of RET-He measures as a diagnostic and therapeutic predictor for improving the management of anemia-associated ESRD. Further prospective cohort multi-centered studies with larger sample sizes from different geographical regions are required to assess the correlation between inter-individual dose variability and anemia outcomes using immature erythrocyte/RET parameters as early biomarkers to obtain the optimal sustained hemoglobin level within the consensus recommendation guidelines.

## Ethical approval and consent to participate

The study was approved by the ethics committee of the Faculty of Medicine, Fayoum University under number (R 330). Written informed consent was obtained from each participant. All study procedures were carried out under the ethical standards of the institutional/and or national research committee and the 1964 Declaration of Helsinki as revised in 2008.

## Consent for publication

Not applicable.

## Funding

Not applicable.

## Author's contributions

**SB:** The concept of the study development, critical revision of the manuscript, and corresponding author to the journal. **KS, AR, EE and MT** contributed scientifically to data and sample collection. **HSh:** Laboratory work. **AE:** Data analysis. All authors contributed to writing the original draft of the manuscript and approved its final version.

## Availability of data and materials

The datasets used and/or analyzed during the current study available from the corresponding author on reasonable request

## Conflicts of interest

All authors declare that they have no conflict of interest.
